# Immune and Metabolic Signatures of COVID-19 Revealed by Transcriptomics Data Reuse

**DOI:** 10.3389/fimmu.2020.01636

**Published:** 2020-06-26

**Authors:** Luiz G. Gardinassi, Camila O. S. Souza, Helioswilton Sales-Campos, Simone G. Fonseca

**Affiliations:** ^1^Departamento de Biociências e Tecnologia, Instituto de Patologia Tropical e Saúde Pública, Universidade Federal de Goiás, Goiânia, Brazil; ^2^Departamento de Análises Clínicas, Toxicológicas e Bromatológicas, Faculdade de Ciências Farmacêuticas de Ribeirão Preto, Universidade de São Paulo, Ribeirão Preto, Brazil

**Keywords:** COVID-19, transcriptomics, inflammation, metabolism, SARS-CoV-2, SARS-CoV, influenza, oxidative phosphorylation

## Abstract

The current pandemic of coronavirus disease 19 (COVID-19) has affected millions of individuals and caused thousands of deaths worldwide. The pathophysiology of the disease is complex and mostly unknown. Therefore, identifying the molecular mechanisms that promote progression of the disease is critical to overcome this pandemic. To address such issues, recent studies have reported transcriptomic profiles of cells, tissues and fluids from COVID-19 patients that mainly demonstrated activation of humoral immunity, dysregulated type I and III interferon expression, intense innate immune responses and inflammatory signaling. Here, we provide novel perspectives on the pathophysiology of COVID-19 using robust functional approaches to analyze public transcriptome datasets. In addition, we compared the transcriptional signature of COVID-19 patients with individuals infected with SARS-CoV-1 and Influenza A (IAV) viruses. We identified a core transcriptional signature induced by the respiratory viruses in peripheral leukocytes, whereas the absence of significant type I interferon/antiviral responses characterized SARS-CoV-2 infection. We also identified the higher expression of genes involved in metabolic pathways including heme biosynthesis, oxidative phosphorylation and tryptophan metabolism. A BTM-driven meta-analysis of bronchoalveolar lavage fluid (BALF) from COVID-19 patients showed significant enrichment for neutrophils and chemokines, which were also significant in data from lung tissue of one deceased COVID-19 patient. Importantly, our results indicate higher expression of genes related to oxidative phosphorylation both in peripheral mononuclear leukocytes and BALF, suggesting a critical role for mitochondrial activity during SARS-CoV-2 infection. Collectively, these data point for immunopathological features and targets that can be therapeutically exploited to control COVID-19.

## Introduction

The outbreak of coronavirus disease 19 (COVID-19), first recognized in Wuhan, China, rapidly became a pandemic of major impact not only on global public health but also on economy and social well-being (1). SARS-CoV-2 infection results in clinical outcomes ranging from asymptomatic status to severe disease and ultimately, death (2). Understanding of the molecular mechanisms underlying the pathology of COVID-19 is required to design effective therapies and safe vaccines. In this context, current investigations have been devoted to biochemical characterization and cellular phenotyping in patients to development of animal models of COVID-19 (3).

Transcriptomics of peripheral blood cells has been a powerful tool to characterize human immune responses to diverse pathogens, including respiratory viruses (4–6). Gene expression profiling by different analytical platforms and sample types revealed that COVID-19 patients exhibit: (i) activation of humoral immunity, hypercytokinemia, apoptosis (7), and dynamic toll like receptor (TLR) signaling (8) in peripheral leukocytes; (ii) induction of interferon stimulated genes (ISGs), chemokines and inflammation in the lower respiratory tract (7, 9, 10). Of importance, the results and interpretation of these data were based on single-gene-level analyses, in which significance of quantitative changes of each gene are calculated separately and they are latter submitted to pathway enrichment analysis. However, the statistical power and sensitivity to identify pathways, or gene modules (computational gene networks), associated with disease phenotypes can be enhanced by the use of non-parametric rank-based tests such as the robust positional framework Gene Set Enrichment Analysis (GSEA) (11). Moreover, interpretation of transcriptional changes during COVID-19 has been primarily evaluated using canonical pathways that do not often reflect human responses. Therefore, we propose alternative strategies to analyze and interpret transcriptomics data, which provide novel insights into immune and metabolic responses during COVID-19.

## Materials and Methods

### Data Collection and Processing

Datasets used in this study included public transcriptomes available at the Genome Sequence Archive (GSA) or human GSA in National Genomics Data Center, Beijing Institute of Genomics (BIG), Chinese Academy of Sciences for RNA-seq data related to SARS-CoV-2 infection (CRA002390 and HRA000143); Gene Expression Omnibus (GEO) for RNA-seq data related to SARS-CoV-2 infection (GSE147507) and microarray data related to SARS-CoV-1 infection (GSE1739) or Influenza A virus (IAV) infection (GSE34205, GSE6269, GSE29366, GSE38900, GSE20346, GSE52428, GSE40012, GSE68310, GSE61754, GSE90732); and ArrayExpress for NanoString nCounter data related to SARS-CoV-2 infection (E-MTAB-8871). DESeq2-normalized counts were used for the RNA-seq dataset CRA002390 (7), while raw read counts for the RNA-seq datasets GSE147507 (9) or HRA000143 (10) were treated and normalized to log_2_ counts per million with EdgeR package for R (12). Normalized data was acquired for NanoString nCounter E-MTAB-8871 (8). Normalized microarray datasets were acquired with OMiCC platform (13). Detailed information about the datasets used in this study are described in Table 1.

**Table 1:** Publicly available datasets used in the study.

**Dataset ID**	**Platform/Technology**	**Virus infection^a^**	**Sample type^b^**	**Sample size (I/C)^c^**	**Data Repository^d^**	**References**
CRA002390	MGI and Illumina/RNA-seq	SARS-CoV-2	PBMC/BALF	3/3	GSA-BIG	(7)
HRA000143	Illumina/RNA-seq	SARS-CoV-2	BALF	8/20	hGSA-BIG	(10)
E-MTAB-8871	NanoString nCounter	SARS-CoV-2	Whole blood	3/10	ArrayExpress	(8)
GSE147507	Illumina/RNA-seq	SARS-CoV-2	Lung tissue	2/2	GEO	(9)
GSE1739	Affymetrix/Microarray	SARS-CoV-1	PBMC	10/4	GEO	(14)
GSE34205	Affymetrix/Microarray	IAV	PBMC	28/12	GEO	(15)
GSE6269	Affymetrix/Microarray	IAV	PBMC	18/6	GEO	(16)
GSE20346	Illumina/Microarray	IAV	Whole blood	19/18	GEO	(17)
GSE29366	Illumina/Microarray	IAV	Whole blood	16/9	GEO	
GSE40012	Illumina/Microarray	IAV	Whole blood	40/18	GEO	(18)
GSE38900	Illumina/Microarray	IAV	Whole blood	16/31	GEO	(19)
GSE52428	Affymetrix/Microarray	IAV	Whole blood	124/17	GEO	(20)
GSE61754	Illumina/Microarray	IAV	Whole blood	66/22	GEO	(21)
GSE68310	Illumina/Microarray	IAV	Whole blood	52/12	GEO	(22)
GSE90732	Illumina/Microarray	IAV	Whole blood	86/22	GEO	(23)

a *SARS-CoV-2, severe acute respiratory syndrome coronavirus 2; SARS-CoV-1, severe acute respiratory syndrome coronavirus 1; IAV, influenza A virus*.

b *PBMC, peripheral blood mononuclear cells; BALF, bronchoalveolar lavage fluid*.

c *(I/C), samples from infected patients/samples from healthy controls*.

d *GSA-BIG/hGSA-BIG, Genome Sequence Archive (GSA)/Human Genome Sequence Archive (hGSA) in National Genomics Data Center, Beijing Institute of Genomics (BIG), Chinese Academy of Sciences https://bigd.big.ac.cn/gsa-human/; GEO, Gene Expression Omnibus https://www.ncbi.nlm.nih.gov/geo/; ArrayExpress, ArrayExpress Archive of Functional Genomics Data https://www.ebi.ac.uk/arrayexpress/*.

### Functional Analyses

Data were analyzed with the positional framework Gene Set Enrichment Analysis (GSEA) (11), using pre-ranked mode, 1,000 permutations and weighted enrichment statistics. The Blood Transcriptional Modules (BTMs) (24) and metabolic pathways annotated in the Kyoto Encyclopedia of Genes and Genomes (KEGG) database (25) were used as gene sets.

To construct the network of BTMs from peripheral blood mononuclear cell (PBMC) transcriptomes, genes were pre-ranked by the Wald test statistics score calculated with DESeq2 package comparing each gene in COVID-19 patients and healthy controls, as described (7). BTMs detected with a false discovery rate (FDR) adjusted *p* < 0.001 were then linked by the number of genes shared between two gene modules.

To perform the BTM-driven meta-analysis between respiratory viruses, gene lists from each dataset were pre-ranked by log_2_ fold change of experimental samples over healthy controls. Gene modules significantly associated with at least 50% of the datasets were selected by a nominal *p* < 0.001 for PBMCs and whole blood. The datasets were not merged at the single-gene-level. Each dataset was composed by a different number of genes and samples, and different types of samples (Table 1). The output of the GSEA provides a normalized enrichment score (NES) for each BTM associated with each dataset. The NES was then compared between datasets selected at the determined cut-off (*p* < 0.001). To enforce confidence in the enrichments, we also retained only the BTMs that were associated with at least 50% of the datasets, independently of infection, sample type and regulation. Metabolic pathways from KEGG database were selected by a FDR adjusted *p* < 0.05 for PBMCs from COVID-19 patients.

For BALF datasets (CRA002390 and HRA000143), genes were also pre-ranked by log_2_ fold change of experimental samples over healthy controls and used as input in pre-ranked GSEA. BTMs and KEGG metabolic pathways were selected by relaxed significance (nominal *p* < 0.05) and consistent up- or downregulation in both datasets. For lung biopsies (GSE147507), one sample from COVID-19 patients shows a distinct read count profile and was considered an outlier as described (26). The remaining sample was used to perform single sample GSEA, in which genes were pre-ranked by log_2_ fold change of the experimental sample over healthy controls.

Networks were visualized and generated with Cytoscape v3.7.2 (27). Heat maps were generated with the package *gplots* for R and hierarchical clustering with the package *amap* for R, using Euclidian distance metric and Ward linkage. The bubble plots were generated with the package *ggplot2* for R. GraphPad Prisma v. 8 was used to perform *t*-tests on NanoString nCounter data and generate bar plots.

## Results

### Modular Transcriptional Network of Peripheral Leukocytes From COVID-19 Patients

**Figure 1:**
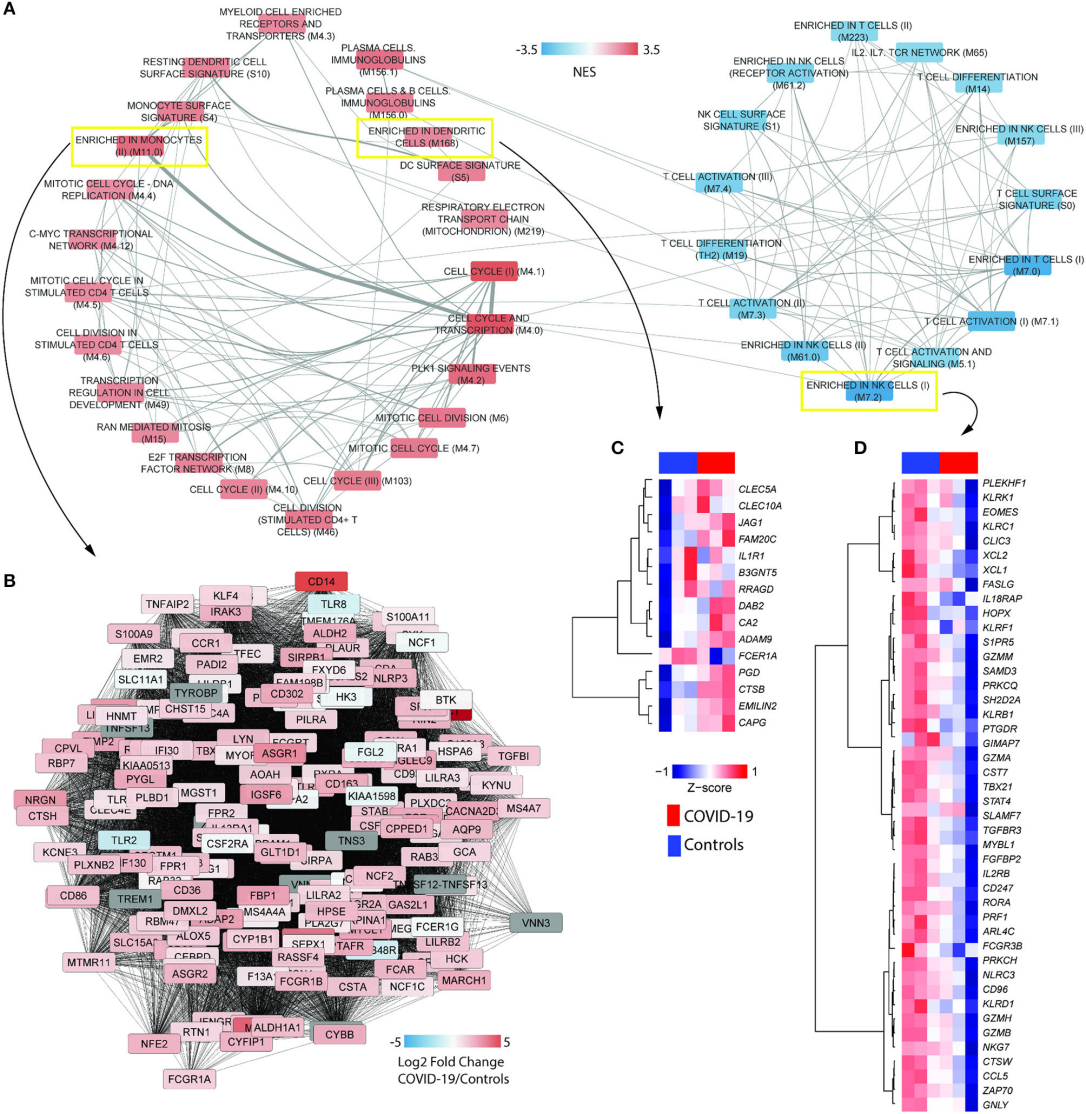
COVID-19 induces the differential activity of gene modules underlying immune cells. **(A)** BTM association with the transcriptional profile of PBMCs from COVID-19 patients (RNA-seq dataset CRA002390) was determined with gene set enrichment analysis (GSEA), with 1,000 permutations and weighted enrichment statistics. The gene list was pre-ranked by Wald statistic scores derived from DESeq2 output. Nodes in the network indicate BTMs reaching a significance of FDR adjusted *p* < 0.001. Colors represent the normalized enrichment scores (NES) of each BTM. Width of edges represent the number of genes shared by two BTMs. **(B)** Representative network of the BTM enriched in monocyte (M11.0). Colors represent log_2_ fold changes of each gene in the transcriptome of COVID-19 patients compared to healthy controls. **(C,D)** Heat maps representing the differential expression signatures of genes enriched in **(C)** dendritic cells (M168) and genes enriched in **(D)** natural killer (NK) cells I (M7.2), between COVID-19 patients and healthy controls.

To evaluate the robustness of our approach, validate previous findings and obtain novel perspectives into immune responses to SARS-CoV-2 infection, we constructed a modular transcriptional network of PBMCs from COVID-19 patients. Genes were pre-ranked by the Wald test statistics score calculated with DESeq2 package [7[, and used as input in pre-ranked GSEA. We interpreted the dynamics in gene expression of COVID-19 patients using the alternative tool to conventional pathways, the BTMs, which were particularly devised to evaluate human immune responses (24). To ensure maximal confidence, we applied a conservative statistical cutoff (FDR adjusted *p* < 0.001) to select significant BTMs (Figure 1A). The transcriptional network captured several cellular characteristics of SARS-CoV-2 infection in peripheral blood, including T and NK cell (Figure 1D) cytopenia (28), and upregulation of cell cycle or genes associated with plasma cells and immunoglobulins (7). In addition, our approach also detected increased signals of monocytes (Figure 1B), dendritic cells (Figure 1C) and of the mitochondrial respiratory electron transport chain in SARS-CoV-2 infection (Figure 1A), suggesting a critical role of metabolic pathways for the immune response of COVID-19 patients.

### Transcriptional Features of SARS-CoV-2 Infection Compared to SARS-CoV-1 and IAV

**Figure 2:**
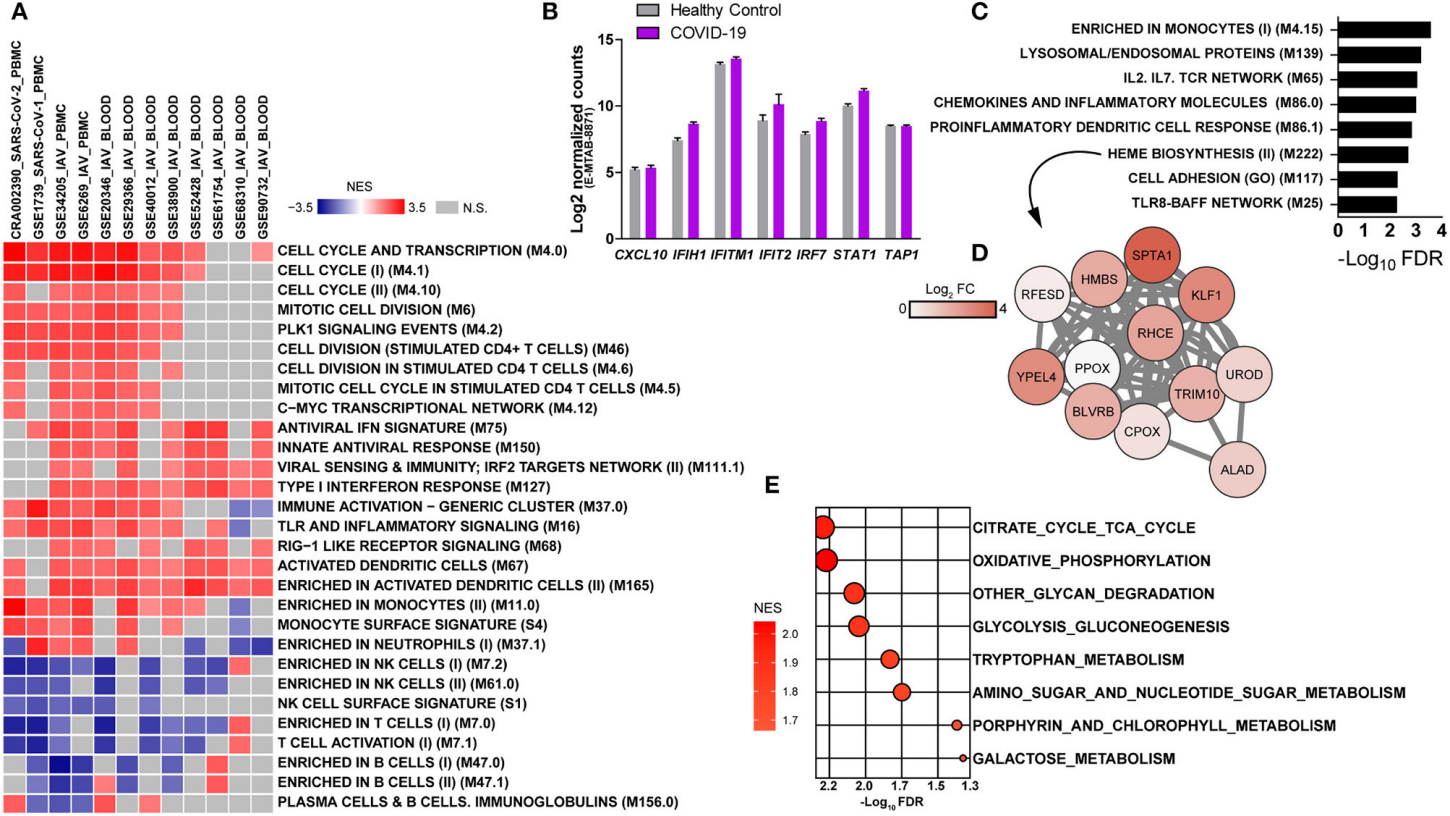
Modular transcriptional profiles of SARS-CoV-2 infection compared to SARS-CoV-1 or IAV. **(A)** The BTM-driven meta-analysis was based on over 600 human transcriptome samples including: SARS-CoV-2 (CRA002390-PBMC), SARS-CoV-1 (GSE1739-PBMC), Influenza (IAV)-PBMC (GSE34205, GSE6269), and IAV-whole blood (GSE29366, GSE38900, GSE20346, GSE52428, GSE40012, GSE68310, GSE61754, GSE90732). Gene lists were pre-ranked by log_2_ fold change of experimental samples over healthy controls and used as input in GSEA, with BTMs as gene sets, 1000 permutations and weighted enrichment statistics. BTMs reaching a significance of nominal *p* < 0.001 and associated with at least 50% of the datasets are shown. Colors represent the normalized enrichment scores (NES), reflecting negative (blue) or positive (red) regulation. Gray color indicates that difference was not significant. Each dataset was specified by ID, virus and sample type in the heat map **(B)** Expression of type I interferon-related genes in whole blood of an independent cohort of COVID-19 patients and analytical platform (E-MTAB-8871) (8). **(C)** BMTs specifically enriched in PBMCs from COVID-19 patients (FDR adjusted *p* < 0.01). **(D)** Representative network of the heme biosynthesis II (M222) module. Colors represent log_2_ fold changes of each gene in the transcriptome of COVID-19 patients compared to healthy controls. **(E)** Metabolic pathways enriched in the transcriptome of PBMCs from COVID-19 patients. Genes were pre-ranked by log_2_ fold change of COVID-19 patients over healthy controls and used as input in GSEA, with KEGG pathways as gene sets, 1,000 permutations and weighted enrichment statistics. Pathways reaching a significance of FDR adjusted *p* < 0.05 are shown. Bubble color is proportional to the normalized enrichment score (NES) and size to the significance, as indicated in the x axis.

To gather further insights on host responses to SARS-CoV-2 infection, the modular transcriptional signature of COVID-19 patients was compared to that of individuals infected with SARS-CoV-1 or IAV. For this, we analyzed 11 additional public transcriptome datasets, spanning over 600 samples from human PBMCs or whole blood. Gene lists from each dataset were pre-ranked by the log_2_ fold changes relative to healthy controls and used as input in pre-ranked GSEA. The statistical cutoff was established at nominal *p* < 0.001, whereas only BMTs present in at least 50% of datasets are shown (Figure 2A). Independently of the cohort, technology to quantify gene expression (RNA-seq or microarray) and type of sample (PBMCs or whole blood), we observed a core transcriptional response that is comparable between infections caused by SARS-CoV-2, SARS-CoV-1, and IAV. This core response includes modules of cell cycle and proliferation, monocytes and dendritic cells. Indeed, the module M67 (dendritic cells) was upregulated in almost all datasets. Of interest, SARS-CoV-1 and IAV infections also induced significant reduction of peripheral T lymphocytes and NK cells. Datasets from IAV infection induced activation of type I interferon/antiviral responses or RIG-1 like receptor signaling, while only SARS-CoV-1 induced significant association to one module, antiviral IFN signature. Data from a different cohort of patients and analytical platform also demonstrated that several genes involved in type I interferon/antiviral responses were not significantly altered in whole blood of COVID-19 patients (Figure 2B). We also evaluated BTMs that were uniquely associated to the transcriptomes from COVID-19 patients, which showed enrichment in immune-related modules and heme biosynthesis (Figure 2C). Data indicates an upregulation of heme biosynthesis in PBMCs from COVID-19 patients (Figure 2D). Because immune responses are tightly connected to metabolic programs (4, 29–31), we explored metabolic pathway enrichment with the KEGG database. In addition to porphyrin metabolism, which shares significant proportion of genes with BTM M222 (heme biosynthesis II), our analysis confirmed the upregulation of glycolysis and gluconeogenesis (7), and detected other pathways such as tricarboxylic acid (TCA) cycle, oxidative phosphorylation, tryptophan metabolism, glycan degradation, nucleotide metabolism and galactose metabolism (Figure 2E).

### Inflammatory and Metabolic Signatures of Lower Respiratory Tracts From COVID-19 Patients

**Figure 3:**
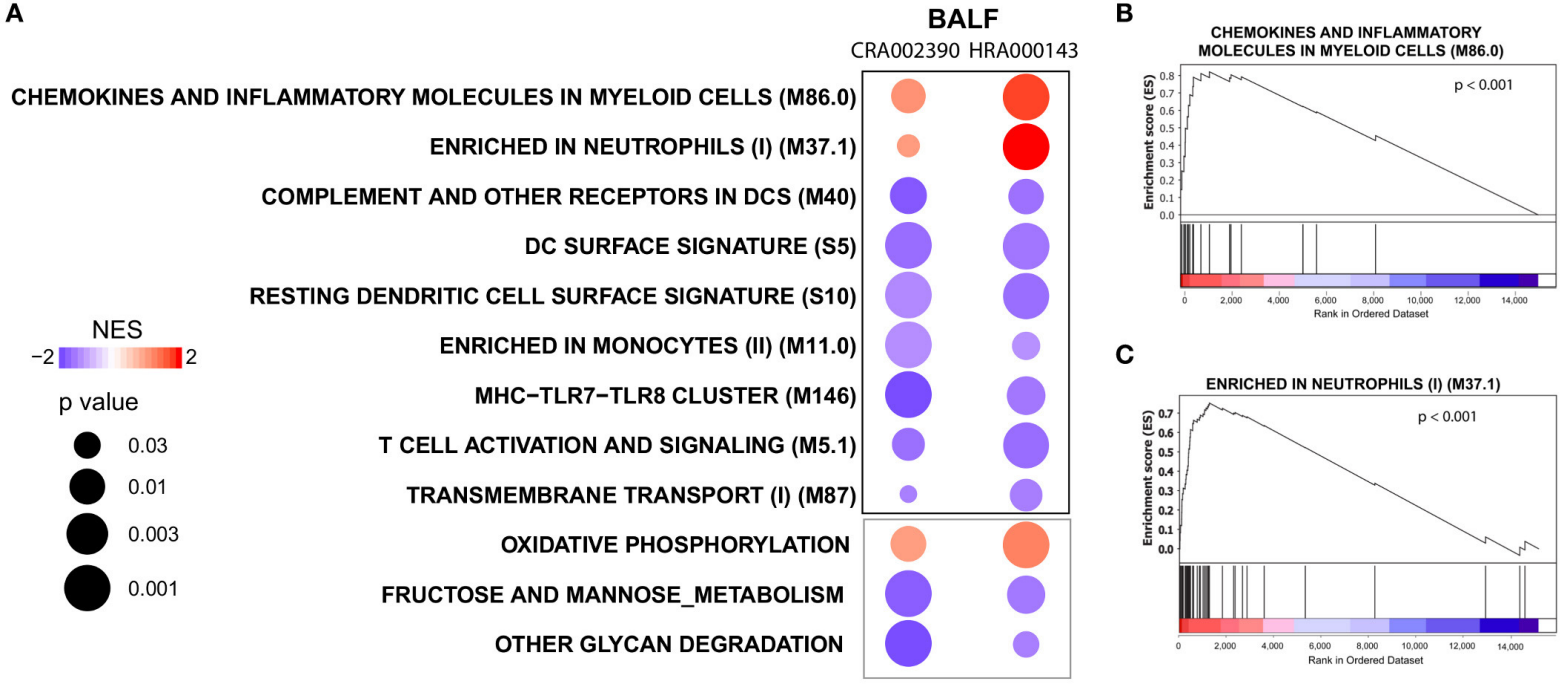
Modulation of immune networks and metabolic pathways in the lower respiratory tract of COVID-19 patients. **(A)** BTM-driven meta-analysis of bronchoalveolar lavage fluid transcriptomes (BALF) (RNA-seq datasets CRA002390 and HRA000143) from COVID-19 patients (7, 10). Gene lists were pre-ranked by log_2_ fold change of experimental samples over healthy controls and used as input in GSEA, with BTMs or KEGG metabolic pathways as gene sets, 1,000 permutations and weighted enrichment statistics. BTMs or metabolic pathways reaching a significance of nominal *p* < 0.05 and consistently regulated in both datasets are shown. BTMs are denoted by the black borders and metabolic pathways by gray borders. Bubble colors represent the normalized enrichment score (NES) regulation and sizes are proportional to the significance of the association. **(B,C)** Enrichment plots for the BTMs chemokines and inflammatory molecules in myeloid cells (M86.0) and enriched in neutrophils (M37.1) from an independent sample of one COVID-19 patient's lung tissue (RNA-seq dataset GSE147507) (9). The gene list was pre-ranked by log_2_ fold change of the experimental sample over healthy controls and used as input in GSEA with the BTMs as gene sets, 1,000 permutations and weighted enrichment statistics.

Because the lung is the primary site of infection and failure of this organ is a severe complication of SARS-CoV-2 infection, we also evaluated immune and metabolic signatures in the lower respiratory tract of COVID-19 patients. For that, we performed a BTM-driven meta-analysis of transcriptomes from samples of bronchioalveolar lavage fluid (BALF) (7). Using a relaxed statistical cutoff (nominal *p* < 0.05), there were nine significant BTMs and three KEGG metabolic pathways that were consistently up or downregulated among both datasets (Figure 3A). BTMs reflect upregulated networks of chemokines and neutrophils, as well as reduced expression of genes related to dendritic cells, monocytes, and T cell activation. We also found consistent upregulation of the modules related to chemokines (Figure 3B) and neutrophils (Figure 3C) in lung tissue data from one COVID-19 patient. Few metabolic pathways were consistently regulated between the BALF datasets, including the upregulation of oxidative phosphorylation and downregulation of fructose and mannose metabolism and other glycan degradation (Figure 3A). None of these metabolic pathways were significantly enriched on the sample of lung tissue.

## Discussion

Here, we used a robust modular transcriptomics approach that captured significant changes of cellular patterns in peripheral blood of COVID-19 patients, including T lymphopenia and reduced numbers of NK cells (28). Several hypothesis have been formulated to explain the lymphopenia during COVID-19, including T cell infection by SARS-CoV-2 (32), or T cell exhaustion (33). In addition, we identified upregulated expression of chemokines and neutrophils in the lung tissue and BALF of COVID-19 patients that support an immunopathological role for these granulocytes (34). These data are in line with findings by Zhou et al. (10), which also suggest higher proportion of neutrophils, activated dendritic cells and activated mast cells via cell deconvolution of BALF transcriptomes. Interestingly, our data suggest increased proportion of monocytes and dendritic cells in the circulation, but not in the BALF. Using single-cell RNA-seq, some studies demonstrated that dendritic cells are indeed reduced in the BALF (35) and there are significant phenotypical alterations of monocytes from COVID-19 patients compared to healthy controls (36).

We demonstrated that compared to SARS-CoV-1 or IAV, SARS-CoV-2 infection fails to induce significant type I interferon responses in PBMCs (Figure 2A) or whole blood (Figure 2B), which corroborates the low concentrations of type I interferon in the circulation of COVID-19 patients (9, 37, 38). These findings contrast with induction of ISG expression in both lung tissue (9) and BALF (10) of COVID-19 patients, while recent studies indicate that type I and III interferons negatively affect the lung epithelium during viral infections (39, 40). The transcriptional response of peripheral leukocytes reflects the systemic adaptations to the inflammatory environment imposed by SARS-CoV-2 infection, whereas type I interferon signaling in peripheral leukocytes might affect immunity in other organs such as the kidneys (41). Importantly, recent data suggest an improvement of patients with uncomplicated COVID-19 treated with interferon-alpha2b (42).

We expect that several factors will contribute to differences in transcriptional profiles of larger cohorts of COVID-19 patients, especially those bearing comorbidities associated with severe disease. Higher expression of angiotensin-converting enzyme 2 (ACE2) has been suggested as a potential mechanism of susceptibility of individuals with comorbidities associated with COVID-19 (43). However, severe disease and death also occur after infection of otherwise healthy individuals, indicating that a series of mechanisms account for the severity of COVID-19. Upregulated expression of genes that coordinate heme biosynthesis has been described in sepsis secondary to pneumonia and suggest a protective mechanism against oxidative stress (44). Hypoxia also modulates the expression of genes coding for proteins that coordinate heme biosynthesis (45). We hypothesize that excessive heme accumulation could amplify pro-inflammatory cytokine production (46, 47) or cause intravascular coagulation (48) and promote pathology during COVID-19.

Strikingly, we observed the modulation of several metabolic pathways in PBMCs and BALF, while oxidative phosphorylation was the only significant metabolic pathway overlapping in both compartments. This suggests a critical role for mitochondrial activity during COVID-19. Many metabolites composing the pathways identified in the current study have been quantified via metabolomics of plasma or serum from COVID-19 patients (49, 50). Mass spectrometry measurements revealed the modulation of pathways such as TCA cycle and fructose and mannose metabolism (50), tryptophan metabolism, glycolysis and gluconeogenesis and others (49). Metabolomics analysis of human PBMCs infected with IAV showed activation of tryptophan metabolism and glycolysis, whereas glucose consumption via hexosamine biosynthesis underlies the cytokine storm promoted by IAV infection (51) and could also affect COVID-19. Taken together, this study demonstrates unappreciated inflammatory networks and metabolic pathways that are associated with COVID-19.

## Data Availability Statement

Publicly available datasets were analyzed in this study. This data can be found here: Genome Sequence Archive (GSA) or human GSA in National Genomics Data Center, Beijing Institute of Genomics (BIG), Chinese Academy of Sciences for RNA-seq data related to SARS-CoV-2 infection (CRA002390 and HRA000143); Gene Expression Omnibus (GEO) for RNA-seq data related to SARS-CoV-2 infection (GSE147507) and microarray data related to SARS-CoV-1 infection (GSE1739) or Influenza A virus (IAV) infection (GSE34205, GSE6269, GSE29366, GSE38900, GSE20346, GSE52428, GSE40012, GSE68310, GSE61754, GSE90732); and ArrayExpress for NanoString nCounter data related to SARS-CoV-2 infection (E-MTAB-8871)/b.

## Author Contributions

LG: selected the data, performed data analysis, interpreted the results, and wrote the manuscript. CS, HS-C, and SF: interpreted the results and critically reviewed the manuscript. All authors contributed to the article and approved the submitted version.

## Conflict of Interest

The authors declare that the research was conducted in the absence of any commercial or financial relationships that could be construed as a potential conflict of interest.
